# Predicted COVID-19 fatality rates based on age, sex, comorbidities and health system capacity

**DOI:** 10.1136/bmjgh-2020-003094

**Published:** 2020-09-09

**Authors:** Selene Ghisolfi, Ingvild Almås, Justin C Sandefur, Tillman von Carnap, Jesse Heitner, Tessa Bold

**Affiliations:** 1 Institute for International Economic Studies, Stockholm University, Stockholm, Sweden; 2 LEAP, Bocconi University, Milan, Italy; 3 Center for Global Development, Washington, DC, USA; 4 Aceso Global, Washington, DC, USA

**Keywords:** public health, SARS

## Abstract

Early reports suggest the fatality rate from COVID-19 varies greatly across countries, but non-random testing and incomplete vital registration systems render it impossible to directly estimate the infection fatality rate (IFR) in many low- and middle-income countries. To fill this gap, we estimate the adjustments required to extrapolate estimates of the IFR from high-income to lower-income regions. Accounting for differences in the distribution of age, sex and relevant comorbidities yields substantial differences in the predicted IFR across 21 world regions, ranging from 0.11% in Western Sub-Saharan Africa to 1.07% for high-income Asia Pacific. However, these predictions must be treated as lower bounds in low- and middle-income countries as they are grounded in fatality rates from countries with advanced health systems. To adjust for health system capacity, we incorporate regional differences in the relative odds of infection fatality from childhood respiratory syncytial virus. This adjustment greatly diminishes but does not entirely erase the demography-based advantage predicted in the lowest income settings, with regional estimates of the predicted COVID-19 IFR ranging from 0.37% in Western Sub-Saharan Africa to 1.45% for Eastern Europe.

**Summary box**

- Given limited testing and vital statistics data, few measures of the COVID-19 infection fatality rate (IFR) exist for developing countries.
- In Europe and North America, measures of COVID-19 IFRs are known to vary by age, gender and comorbidities.
- Existing model-based estimates for the developing world have not fully accounted for these factors in predicting IFRs.
- Using variation in demographics, comorbidities and health system capacity, we predict COVID-19 IFRs for 187 countries, ranging from 0.43% in Western Sub-Saharan Africa to 1.45% in Eastern Europe.
- Despite lower measured health system capacities, predicted IFRs for most of Sub-Saharan Africa nonetheless remain well below IFRs for high-income countries, while Eastern Europe is predicted to fare particularly poorly.
- Policy-makers in low-income countries should be cognizant that any demographic advantages with respect to COVID-19 fatality rates are likely to be partially offset by disadvantages in health system capacity.

## Introduction

Key policy decisions for COVID-19 containment hinge on its infection fatality rate (IFR). Data from the hardest-hit countries show that the IFR varies by sex, age and certain comorbidities, suggesting a method to extrapolate estimates to new contexts with limited data infrastructure.^1–7^ In this article, we combine recent estimates of the sex-specific and age-specific IFR from France with data on comorbidities conditional on death with COVID-19 in Italy to calculate the inverse: an IFR conditional on sex, age and comorbidity (cIFR). We apply these estimates to the distribution of sex, age and relevant morbidities for 187 countries from the Global Burden of Disease (GBD) data set.^7^ Results reveal substantial differences across 21 world regions, with demographics-based IFR predictions ranging from 0.11% in Western Sub-Saharan Africa to 1.07% for high-income Asia Pacific. Despite the comparatively low IFR estimates our model predicts for the lowest income regions, these IFR estimates are appreciably higher than other recent estimates for the same areas.^8^

We understand these predicted IFRs as lower bounds on mortality in low- and middle-income countries, since they are derived implicitly assuming access to advanced healthcare. To account for the likelihood of higher fatality rates in under-resourced health systems, we adjust the predicted IFRs for differences in the relative odds of infection fatality from childhood respiratory syncytial virus (RSV) between world regions as a proxy for local capacity to treat viral respiratory illnesses. This adjustment greatly diminishes, but does not entirely erase, the demography-based advantage predicted in the lowest income settings, with regional estimates of the predicted COVID-19 IFR ranging from 0.43% in Western Sub-Saharan Africa to 1.45% for Eastern Europe.

## Predicting the cIFR

Here we outline the calculation of our benchmark: the predicted cIFR status, starting from the IFR estimates by age and sex reported in Salje *et al* 4 for France. The latter are, to our knowledge, the most recent peer-reviewed IFR estimates for COVID-19 which report variations for all age brackets and differentiate by sex. They are lower than earlier figures from Walker *et al* 9, particularly among younger age groups, but are quite similar in the highest age brackets.

The core assumption behind our approach is that variation in the IFR *within* France by age, sex and comorbidity can be used to predict the variation in IFRs *across* countries based on their age, sex and comorbidity distributions. To date these are the key factors that have well studied, statistically and clinically significant associations with COVID-19 severity and death. Importantly, we do not require that the underlying distributions of age, sex or comorbidities are similar between France and other countries in our sample; on the contrary, differences across countries in these distributions will drive the variation in predicted IFRs. We now demonstrate our method to extricate from the French age and sex-specific IFRs that part which we claim is portable across contexts: the probability of dying (d) given infection from COVID-19 (I) and the age (a), sex (s), and comorbidity status (c) of patients, that is, P(d|c;I,a,s). We term this the cIFR and use subscripts for notational convenience, so that

$$cIFR=P_{Ias}\left(d|c\right)$$

Applying Bayes’ rule, we can recover this cIFR by relating it to the ratio of comorbidity prevalence among COVID-19 fatalities relative to COVID-19 infections (conditional on age and sex) and age and sex-specific IFRs:

(1) $$cIFR=P_{Ias}\left(d|c\right)=\frac{P_{Ias}\left(c|d\right)}{P_{Ias}\left(c\right)}P_{Ias}\left(d\right)$$

We now discuss how we measure each of these probabilities.

(1) $P_{Ias}\left(c|d\right)$ denotes the probability of comorbidity status given death of COVID-19, age and sex. We rely on the assumption that this probability is independent of age and sex, $P_{Ias}\left(c|d\right)\approx P\left(c|d,I\right)$, which is supported by data from New York City. (As shown in online supplemental figure 1, data from New York City indicate that among those who die from COVID-19, the share that has any comorbidity is stable across age groups and very similar for both.) We calculate P(c|d,I), using the Italian Istituto Superiore della Sanità reports on the number of comorbidities conditional on COVID-19 death.^10^ The choice to combine data from France and Italy was motivated by the fact that the latest published estimates of mortality by age and gender come from France, while reliable data on comorbidities among COVID-19 deaths are available for Italy but not France. Given our assumption that the cIFR is portable across contexts (with the same health system capacity), countries with the same comorbidity and sex distribution at each age should have the same age-specific IFR. We show in theonline supplemental figure 1 that France and Italy are similar in terms of comorbidity and sex distributions for a given age, and that the age-specific IFR estimates for the two countries (reported in Salje *et al* 4 and Ferraro *et al* 11) are very close. Thus by equation (1), the two countries should also have the same prevalence of comorbidities among their COVID-19 fatalities at each age.

10.1136/bmjgh-2020-003094.supp2 Supplementary data

(2) $P_{Ias}\left(c\right)$ denotes the presence of underlying conditions given infection, age and sex. We assume $P_{Ias}\left(c\right)\approx P\left(c|a,s\right)$ and take the probability of having any COVID-19-relevant comorbidity by age and sex in France from the GBD data set. This assumption would be violated if the pool of infected systematically differs from the general population. Recent evidence from the USA suggests that comorbidities are as present among the infected as in the general population.^12^ Furthermore, data from Italy show attack rates above 50% in some provinces. This, together with the absence of widespread immunity^11^ further supports this claim. Note that for simplicity we rely on an indicator for any COVID-19-relevant comorbidity, although the type, number and combination of different diagnoses are likely to affect the cIFR. The comorbidities considered relevant for COVID-19 by Clark *et al* 7 are the following: cardiovascular diseases, chronic kidney diseases, chronic respiratory diseases, chronic liver disease, diabetes mellitus, cancers with direct immunosuppression, cancers with possible immunosuppression, HIV/AIDS, tuberculosis, chronic neurological disorders, sickle cell disorders.

(3) $P_{Ias}\left(d\right)$ denotes the sex and age-specific IFRs from Salje *et al* 4 which come from France.

**Figure 1:**
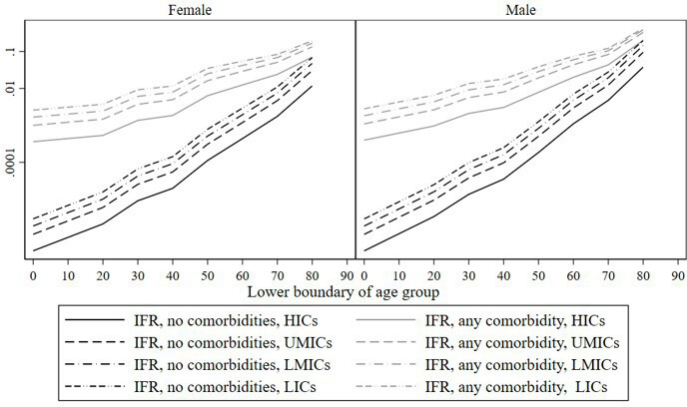
cIFRs, adjusted for health system capacity, by country income group (log scale). cIFRs, infection fatality rates conditional on age, sex and comorbidity; HICs, high-income countries; LICs, low-income countries; LMICs, lower middle-income countries; UMICs, upper middle-incomecountries.

With these ingredients, we can calculate the cIFR assuming healthcare levels similar to high-income countries (HICs) in (1), which we find to be an increasing and non-linear function of both age and comorbidity (figure 1 and table 1, labelled ‘HIC’). For those without a comorbidity, the cIFR is effectively zero and flat up to the age of 50, and then increases roughly 20-fold between 50–59 and 70–79 years (from 0.01% to 0.17% for women and from 0.02% to 0.48% for men). With a comorbidity, the pattern is similar, but because the cIFR is already higher at younger ages, the age gradient is flatter, roughly doubling the cIFR for each decade above age 50. The difference in the cIFR between patients with and without comorbidities is large but declines rapidly with age. Finally, the female cIFR is lower than the male cIFR for each age and comorbidity status.

**Table 1:** cIFRs, adjusted for health system capacity, by income group (percentage points)

Comorbidity	LIC				LMIC			
	Females		Males		Females		Males	
	0	>0	0	>0	0	>0	0	>0
Age, years								
0–19	0.0003	0.2583	0.0003	0.2807	0.0002	0.1670	0.0002	0.1825
20–29	0.0016	0.3727	0.0025	0.6551	0.0010	0.2435	0.0016	0.4324
30–39	0.0066	0.9167	0.0098	1.3538	0.0043	0.6080	0.0063	0.9127
40–49	0.0142	1.1632	0.0249	1.8018	0.0092	0.7912	0.0161	1.2563
50–59	0.0783	3.4838	0.1263	3.8523	0.0511	2.4923	0.0832	2.8623
60–69	0.2917	5.4639	0.7042	7.4230	0.1941	4.1954	0.4768	5.9842
70–79	1.0952	8.3357	2.7756	12.1281	0.7466	6.8290	1.9421	10.4667
80+	6.7997	19.3879	19.9064	42.2803	4.7666	16.7857	14.4353	38.4637

cIFRs, infection fatality rates conditional on age, sex and comorbidity; LIC, low-income country; LMIC, lower middle-income country.

**Table:** 

Comorbidities	UMIC				HIC			
	Females		Males		Females		Males	
	0	>0	0	>0	0	>0	0	>0
Age, years								
0–19	0.0001	0.1001	0.0001	0.1098	0.00004	0.0361	0.00004	0.0397
20–29	0.00060	0.1470	0.00095	0.2631	0.00021	0.0534	0.00034	0.0963
30–39	0.0025	0.3713	0.0038	0.5645	0.0009	0.1364	0.0014	0.2100
40–49	0.0055	0.4928	0.0097	0.7985	0.0020	0.1847	0.0035	0.3057
50–59	0.0308	1.6196	0.0505	1.9255	0.0112	0.6353	0.0185	0.7865
60–69	0.1188	2.9143	0.2959	4.3776	0.0438	1.2395	0.1105	2.0008
70–79	0.4659	5.0878	1.2381	8.3111	0.1749	2.3906	0.4755	4.3483
80+	3.0436	13.3843	9.4946	32.8492	1.1708	7.0568	3.7759	19.9581

Table reports the health-system adjusted cIFR derived in Section Predicting the cIFR and online supplementary appendix A1.

HIC, high-income country; UMIC, upper middle-income country.

10.1136/bmjgh-2020-003094.supp1 Supplementary data

**Figure 2:**
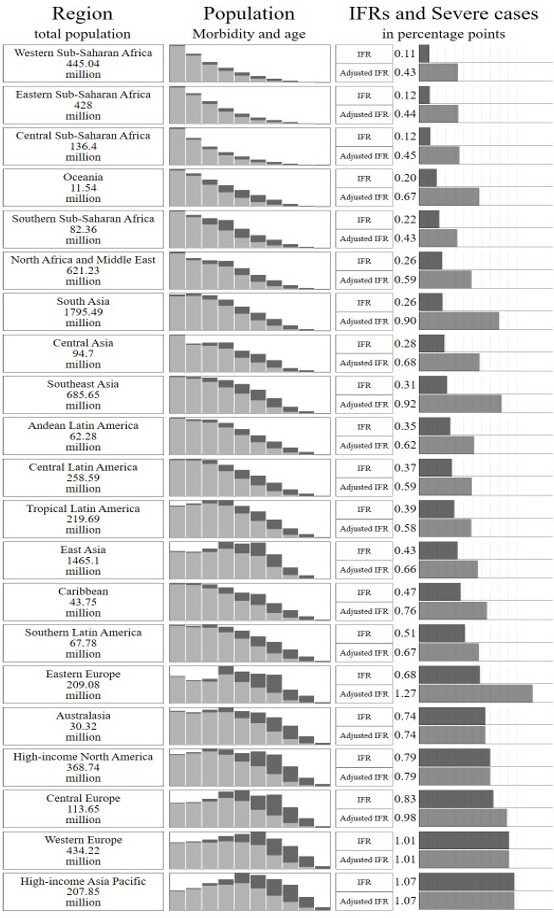
Infection fatality ratio (IFR) by world region. Column 1 states total population in millions for each region. Column 2 reports population by 10-year age groups and by number of comorbidities (light grey: 0 comorbidity; dark grey: any comorbidity); the height of the graphs is proportional to the number of people in the most populous age group. Column 3 reports (a) regional IFRs calculated as an average of the IFRs conditional on age, sex and comorbidity weighted by the proportion of the population in each age, sex and comorbidity group and (b) regional IFRs adjusted for health system capacity (see Section Adjusting for differences in health system capacity).

We integrate the cIFR over each country’s sex, age and comorbidity distribution to obtain a country-specific average IFR. Figure 2 shows our main results, aggregated by 21 world regions (we display the unaggregated results in online supplemental figure 2). We find substantial variation in predicted IFRs across regions—by a factor of 10 between the highest (high-income Asia Pacific with an IFR of 1.07%) and the lowest (Western Sub-Saharan Africa with an IFR of 0.11%). The variation is systematic, as low-income regions have lower predicted IFRs than high-income regions. Demography is a key driver of these results: age distributions vary substantially across regions, with Sub-Saharan Africa and Oceania having the youngest and richer regions having the oldest populations. Regional variation in comorbidities also helps explain variation in predicted IFRs across regions: high-income regions display more comorbidities among the elderly than low-income settings, while the reverse is true among the young and middle-aged segments of the population. Finally, because the IFR is always lower for women than for men, variation in sex imbalances in the highest age brackets (tilted toward women everywhere) also contributes to variation in the average IFR.

10.1136/bmjgh-2020-003094.supp3 Supplementary data

## Adjusting for differences in health system capacity

We interpret our predicted IFR estimates as lower bounds on the true probability of dying from COVID-19 in low and middle-income settings, as data on fatalities come from countries with advanced health systems. Health system weaknesses in lower income settings likely imply that a larger proportion of severe COVID-19 cases result in death due to suboptimal medical care, and this will likely diminish the demographic advantages of low-income countries (LICs). To account for this, we adjust our IFR estimates for health-system strength based on a region’s demonstrated capacity to prevent fatalities from viral lung infections. We derive this adjustment from comparative regional hospital case fatality rates for RSV among children aged 0–59 months.

We chose this demographic to derive our health system capacity measure because restricting attention to this age bracket approximately purges the RSV IFRs of cross-country variation in the distribution of ages, comorbidities (as children under five have very low burdens of chronic diseases such as hypertension, kidney disease or other conditions of organ degradation) and sex (as sex ratios under 5 years are more balanced than for older groups). With nearly equivalent age, sex and comorbidity rates in this demographic, we take remaining cross-country variation in the IFR for RSV to be attributable principally to health system capacity. We choose RSV acute lower respiratory infection (ALRI) as a proxy for COVID-19 as they are viral lower respiratory infections with overlapping symptoms. Like COVID-19, RSV usually causes mild symptoms, but occasionally develops into a life-threatening illness. As with all viruses, neither is treatable with antibiotics, and, until COVID-19, RSV was unique among the major organisms that cause death from respiratory tract infections to have neither any vaccine nor recognised treatment.^13 14^

Normalising the IFR for childhood RSV in HICs to 1, we apply the ratio of these IFRs between regions to scale up our demography-adjusted and comorbidity-adjusted IFR predictions. Unfortunately, we lack country-level IFR estimates. However, Shi *et al* 15 provide data from which RSV IFRs for severe cases can be inferred by World Bank income level: HICs, LICs, lower middle-income countries (LMICs) and upper middle-income countries (UMICs). The ratios of the IFRs for children hospitalised with RSV between HICs and LICs, LMICs and UMICs from this data are 8.54, 5.45 and 3.23, respectively. While we assume that all severe cases warranting hospitalisation obtain it in HICs, this is not necessarily the case in other income groups, and thus these relative hospital fatality ratios require an adjustment to become infection fatality ratios. We take this adjustment from Wang *et al*,^16^ from which the relationship between hospital case fatality rates and IFRs can be mapped for LMICs and HICs for childhood influenza, another comparable respiratory virus. Using this mapping, we translate our RSV IFRs specifically among hospitalised children into IFRs among all severe cases, which are estimated to have ratios to HIC IFRs of 7.40, 4.72, 2.80 for LICs, LMICs and UMICs, respectively. Taking these ratios as ORs rather than risk ratios (to maintain coherent probability bounds), we rescale the predicted cIFRs by these region-specific adjustments to calculate a cIFR conditional on regional health system capacity (see online supplementary appendix A1 for details).

Adjusting for health system capacity increases the cIFR in poorer regions by almost an order of magnitude (figure 1 and table 1). At ages 60 and below, the cIFR is increased by a factor of 6–7 in LICs, by a factor of 4 in LMICs and by a factor of 2–3 in UMICs. For older ages, the increase in the cIFR is less stark, but the adjusted cIFR is still two to four times as large as the unadjusted one. Lower health system capacity thus both increases the cIFR at each age and comorbidity status and flattens its age gradient.

With this health system-adjusted cIFR in hand, we recalculate the country-specific IFRs (and add them to figure 2 and online supplemental figure 2). The health system strength adjustment starkly increases the predicted COVID-19 IFRs for the lowest income regions, nearly though not entirely erasing their demographic advantages: the predicted IFRs double on average in UMICs, almost triple in LMICs and increase by a factor of 3.7 in LICs. As examples, IFRs increase from 0.13% to 0.44% in Sub-Saharan Africa, from 0.39% to 0.73% in Latin America and from 0.31% to 0.73% in South and Central Asia. Eastern Europe is predicted to have particularly high IFRs (1.43%), as it is characterised by an ageing population, high prevalence of comorbidities at a given age and low predicted health system capacity based on its income levels.

Our method of accounting for differences in health system capacity is crude in that we currently only have indicative numbers for RSV ALRI by income group, rather than national-level adjustments. However, the wide gap in childhood respiratory tract IFRs of between 2.8-fold and 7.4-fold between income groups has implications for COVID-19 IFRs that are too large to ignore.

## Validating the predictions with serological studies from random samples

We can test the validity of our core assumption, namely, that variation in age, sex and comorbidity distributions as well as health system capacity explain differences in IFRs across countries by comparing our predicted IFRs to independently measured IFRs. For this exercise, we consider all studies reporting either IFRs or infection rates for populations with available COVID-19 fatalities, which were listed in the systematic review by Meyerowitz-Katz and Merone^17^ or retrieved through an online search on July 2. Out of the 32 studies selected in this way, 6 studies measure infection rates by testing for seroprevalence of COVID-19 antibodies in population-based random samples. We judge this to be the best method of estimating infection rates and thus IFRs, because random sampling is required to be truly representative, and antibody seroprevalence indicates all cumulative cases, whereas ‘swab’ tests only detect current cases. We thus compare our predicted IFRs first and foremost to the estimates in these six studies. While five of the six random sample studies are located in HICs, one is from an UMIC, allowing for validation of the health system-adjusted IFRs constructed in the previous section. In a second step, we use all published IFR estimates in the comparison, including those which use convenience samples, adjusted case fatality rates (CFRs) or ‘swab’ tests.

**Figure 3:**
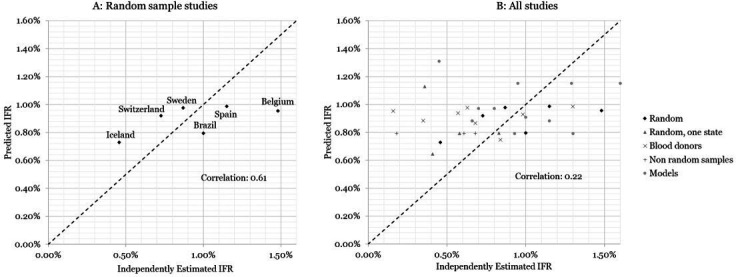
Validation with independently estimated infection fatalityrates (IFRs). (A) Random sample studies, representative of large proportion of country’s population. (B) All studies included in Meyerowitz-Katz and Merone 17 or found through online search.

The results are presented in figure 3A, where we plot the independent IFR estimates for the six random sampling studies in different countries on the horizontal axis against our predicted IFRs—using the health system-adjusted IFRs from the previous section—on the vertical axis. The independent estimates and our predictions are reported in table 2. Comparing our estimates to these studies, we find a correlation of 61%, demonstrating that our method can successfully predict a considerable portion of the cross-country variation in IFRs. We note that Switzerland^18^ and Sweden^19^ are close to the 45° line, as are the estimates from Spain^20^ and Iceland,^21^ which have been acknowledged to be well designed, randomised data collection efforts. For Brazil,^22^ which tests the validity of our approach outside of high-income health systems, the health system-adjusted IFR also closely matches the independently estimated IFR, while the crude IFR is substantially lower at 0.40% (consistent with our expectation that failing to adjust for health system capacity provides a lower bound on the true IFR outside of HICs). Belgium,^23^ on the other hand, has a very high IFR relative to our predicted number, but this source counts all suspect deaths in nursing homes as COVID-19 deaths (as reported in https://www.bbc.com/news/world-europe-52491210), yielding the highest IFR among the included studies.

**Table 2:** Independently estimated IFRs and predicted IFRs adjusted by health system capacity

	Sampling	Location	Income group	Independently estimated IFR	Predicted IFR		Sampling	Location	Income group	Independently estimated IFR	Predicted IFR
	Random	Sweden	HIC	0.87%	0.98%		Blood donors	The Netherlands	HIC	0.98%	0.93%
	Random	Belgium	HIC	1.48%	0.96%		Blood donors	Denmark	HIC	0.57%	0.94%
	Random	Iceland	HIC	0.46%	0.73%		Facebook	Santa Clara, USA	HIC	0.18%	0.79%
	Random	Switzerland	HIC	0.73%	0.92%		Shoppers	New York State, USA	HIC	0.68%	0.79%
	Random	Spain	HIC	1.15%	0.99%		Shoppers	New York City, USA	HIC	0.61%	0.79%
Random	Brazil	UMIC	1.00%	0.80%		SIR	France	HIC	0.80%	0.97%
Random, local	Brazil, Rio Grande do Sul	UMIC	0.83%	0.80%		Adjusted CFR	USA	HIC	1.30%	0.79%
Random, local	Indiana, USA	HIC	0.58%	0.79%		Adjusted CFR	UK	HIC	1.00%	0.91%
Random, local	Iran	UMIC	0.41%	0.65%		Adjusted CFR	Beijing, China	UMIC	1.15%	0.88%
Random, local	Germany	HIC	0.36%	1.13%	Adjusted CFR	Italy	HIC	1.60%	1.15%
Blood donors	Czech Republic	HIC	0.68%	0.87%		Travellers	Japan	HIC	0.45%	1.31%
	Blood donors	Slovenia	HIC	0.16%	0.95%		Travellers	China	UMIC	0.66%	0.88%
	Blood donors	Spain (2)	HIC	1.30%	0.99%		Excluding mortality	Italy (2)	HIC	0.95%	1.15%
	Blood donors	Sweden (2)	HIC	0.63%	0.98%		Model	Northern Italy	HIC	1.29%	1.15%
	Blood donors	Luxembourg	HIC	0.84%	0.75%		Model	France (2)	HIC	0.70%	0.97%
	Blood donors	Wuhan, China	UMIC	0.35%	0.88%		PFR	New York City, USA	HIC	0.93%	0.79%

The table lists all independently estimated IFRs retrieved in an internet search on July 2 and listed in Meyerowitz-Katz and Merone 17 reporting type of study, location and income region. It compares the independent results with our predicted health system-adjusted IFR.

CFR, case fatality rate; HIC, high-income country; IFR, infection fatality rate; PFR, population fatality rate; SIR, susceptible, infectious, recovered model; UMIC, upper middle-income country.

Figure 3B reports the results from a comparison with all the same studies listed in Meyerowitz-Katz and Merone^17^ plus four additional random seroprevalence studies representative at subnational level. Twenty-six studies come from HICs and six from UMICs. The estimates displayed in this panel are much more noisy, including wide variations within single countries. Nonetheless, our method does retain a positive correlation, although a lower one, even with these measured IFRs.

Note that we lack coverage for LICs in this validation exercise. The lack of representative seroprevalence studies and COVID-19 mortality data to estimate IFRs in such contexts is a key motivation for this study and highlights the need for modelled predictions. For example, we are aware of two serological studies measuring prevalence rates from countries in Sub- Saharan Africa: one based on a representative sample of Nampula, Mozambique,^24^ and another of Kenyan blood donors.^25^ However, fatality data appear unreliable: even attributing all recorded deaths from COVID-19 in Mozambique and Kenya (6 and 154 total deaths, respectively) to the surveyed regions of Nampula and Nairobi, the estimated IFRs would be disproportionately low at 0.018% and 0.028%.

## Conclusion

Our results illustrate the possibility of predicting COVID-19 IFRs with a methodology that (1) uses information readily available for most of the world— namely age and comorbidity distributions as well as proxies for health system capacity, (2) relies on parsimonious and transparent assumptions and (3) appears broadly consistent with the limited set of IFRs generated from random COVID-19 testing. Although we produce estimates at national level, subnational variability in distributions of comorbidities, age and sex may be important enough to require IFR estimations at subnational level. A merit of our approach is its portability to any community level where comorbidity, sex and age distributions and health system capacity (compared with France) are known.

While our calculations including adjustments for health system strength still suggest somewhat lower IFRs in the least developed economies than in the most advanced economies, our estimates are significantly higher than IFRs used in other recent COVID-19 forecasts for Africa,^8^ and middle-income countries.^9^ In the absence of widespread testing or reliable vital registration systems, transparent calculations of likely IFRs provide an important input into optimal policy design under extreme uncertainty, particularly as the pandemic expands into new geographies and/or a second wave of infections arrives.
