# Supplementary material for: Predicted COVID-19 fatality rates based on age, sex, comorbidities and health system capacity
Source: BMJ Glob Health. 2020 Sep 9;5(9):e003094. doi: 10.1136/bmjgh-2020-003094 (PMC7482102; doi:10.1136/bmjgh-2020-003094)

A: IFRs Estimated for Italy and France

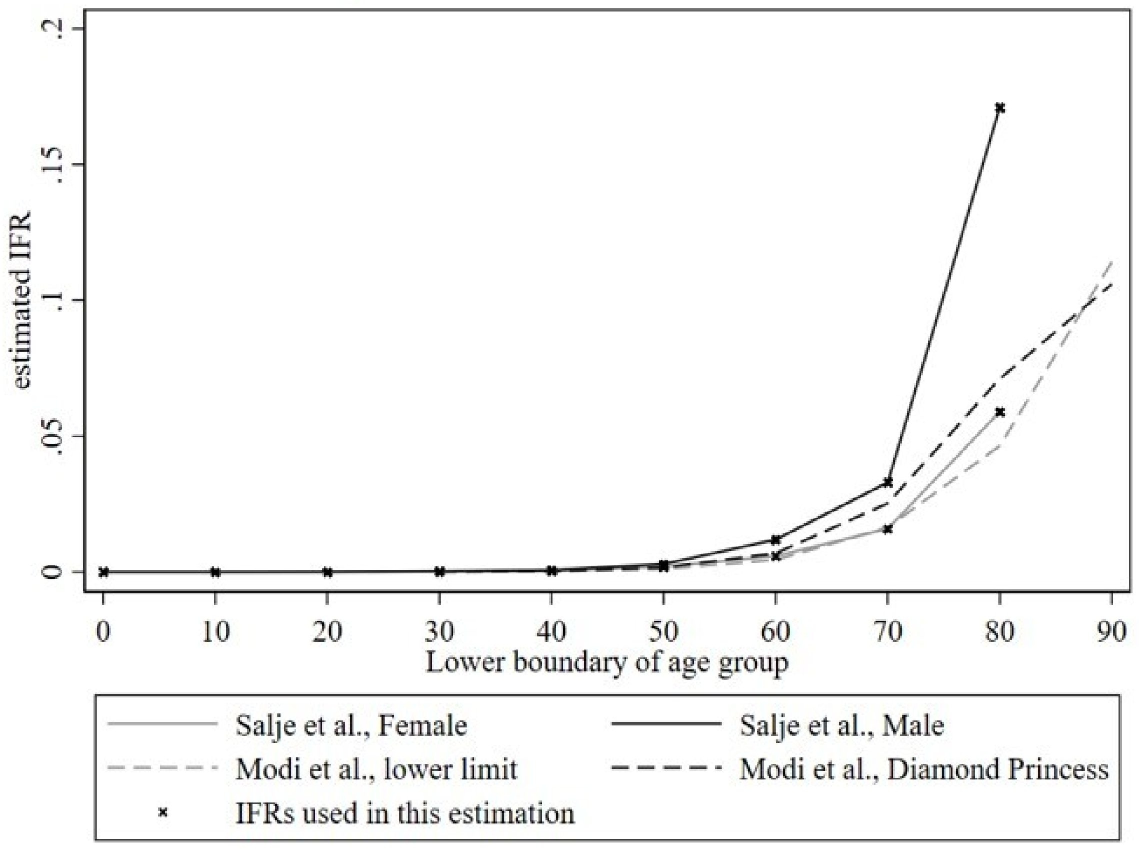

B: Share of population with no comorbidities and sex ratio in Italy and France

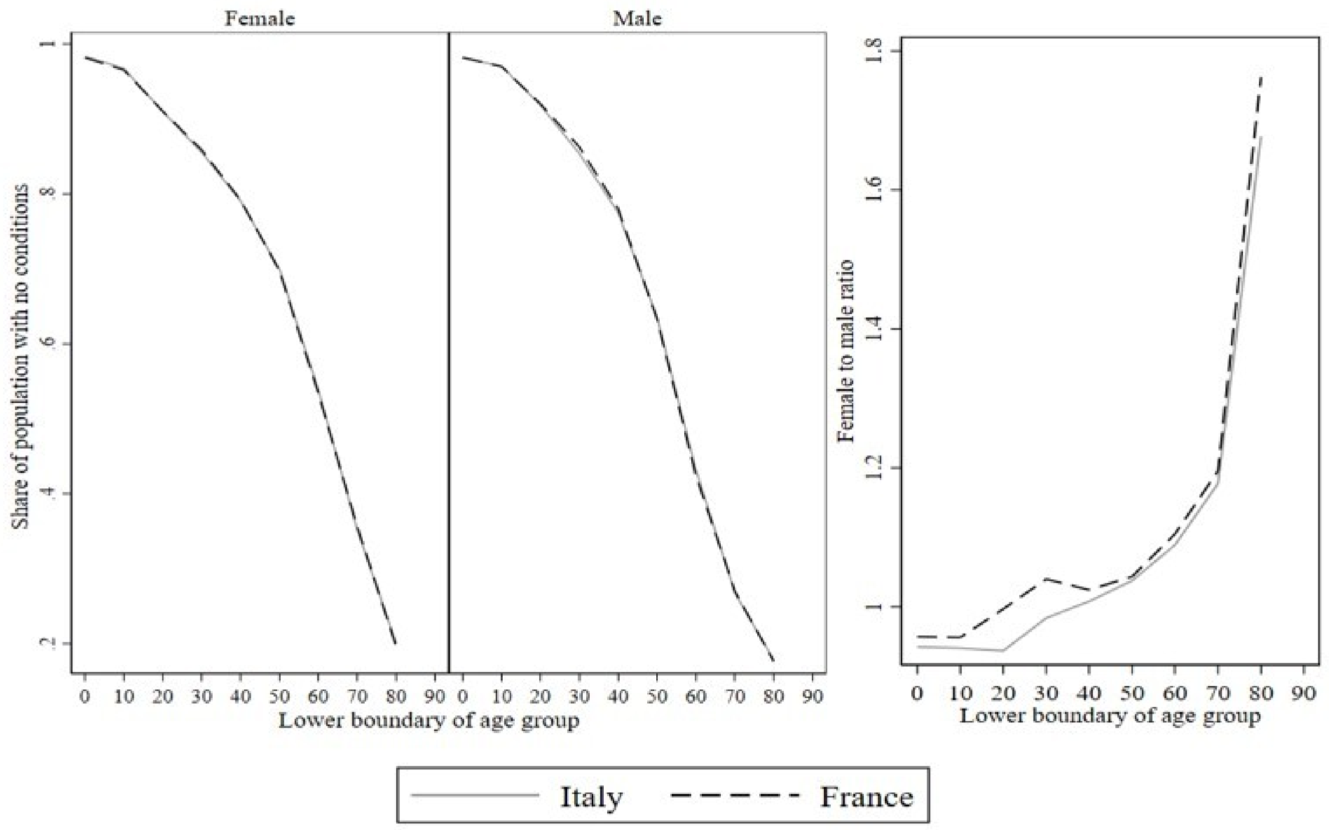

C: Comorbidities among COVID-19 fatalities, Italy and NYC

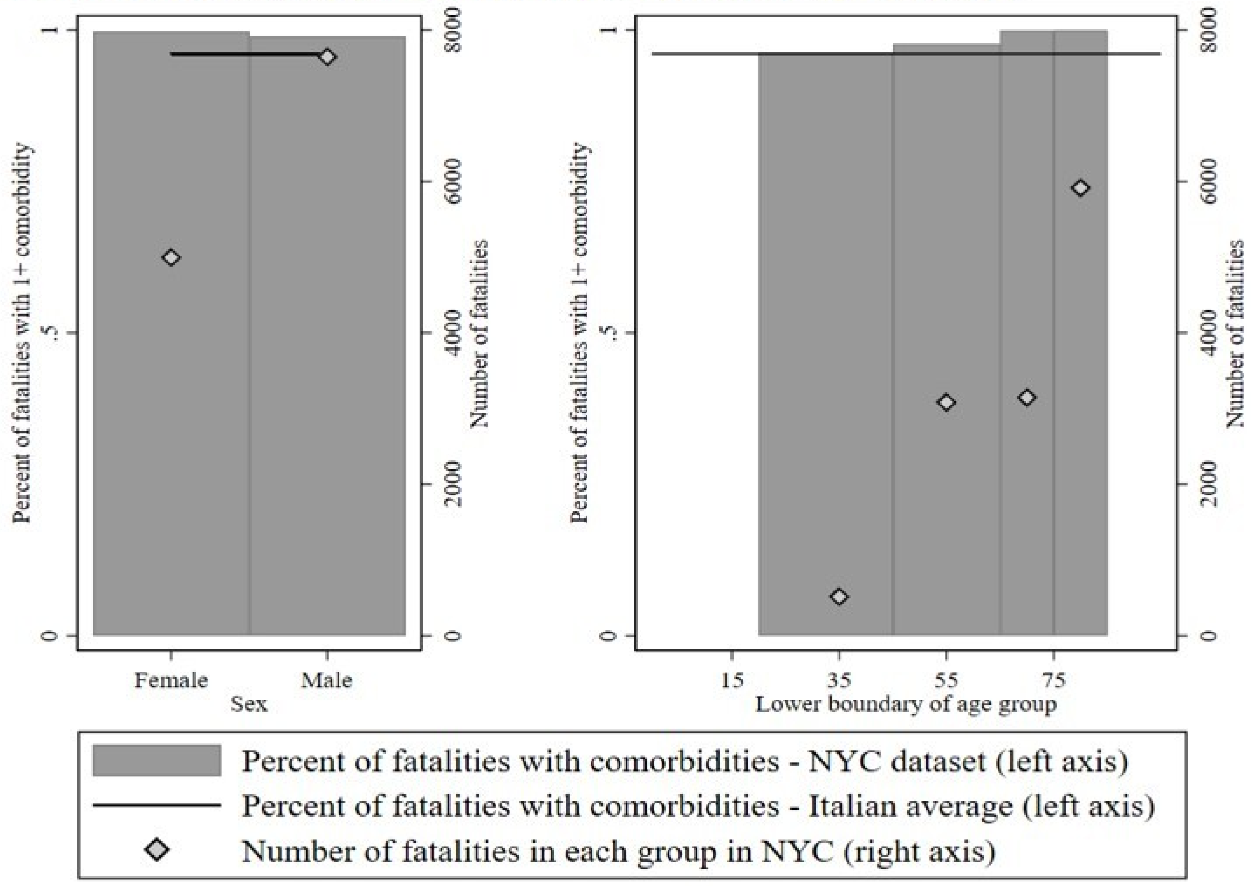

Supplement: Supplementary data [file bmjgh-2020-003094supp002.pdf]
