# Supplementary material for: Predicted COVID-19 fatality rates based on age, sex, comorbidities and health system capacity
Source: BMJ Glob Health. 2020 Sep 9;5(9):e003094. doi: 10.1136/bmjgh-2020-003094 (PMC7482102; doi:10.1136/bmjgh-2020-003094)
Supplement: Supplementary data [file bmjgh-2020-003094supp001.pdf]

Appendix A.1. Health system capacity adjustment

We now explain how to obtain the cIFR adjusted by regional health system capacity in world income group j, where j is either high-income countries (HICs), upper middle-income countries (UMICs), lower middle-income countries (LMICs), or low income countries (LICs).

In a first step, we decompose the severity of COVID-19 infections into two states: (i) having COVID-19 so severe that one would warrant hospitalization and (ii) having a mild version of COVID-19, which does not warrant hospital care. This decomposition is done because we assume – as in [5] and [9] – that mild cases are self-limiting, i.e., will resolve without medical treatment, and thus apply the health system adjustment only to severe cases. The cIFR in income group inc_j can thus be written as

$$P_{Ias}(d|c, inc_j) = P_{Ias}(d|severe, c, inc_j) * P_{Ias}(severe|c, inc_j)\,. \qquad \text{(A.1)}$$

Central to estimating the quantities on the right-hand side are three steps.

1) To obtain $P_{Ias}(severe|c, inc_j)$ we posit, similar to the core assumption in Section 2, that the probability – conditional on age, sex and comorbidity status – of a severe case is constant across contexts, i.e. $P_{Ias}(severe|c, inc_j) = P_{Ias}(severe|c)$. As we note in Section 2, age, sex and comorbidities are the key correlates that have well studied statistically and clinically significant associations with COVID-19 severity and death, and conditioning on them should remove most of the cross-country variation in the IFR. The health system adjusted cIFR for a country in income group inc_j thus simplifies to

$$P_{Ias}(d|c, inc_j) = P_{Ias}(d|severe, c, inc_j) * P_{Ias}(severe|c)\,. \qquad \text{(A.2)}$$

We thus posit that the probability of developing severe COVID-19 does not depend on a country's health system capacity. This is clearly conservative, as the quality of the health system may itself determine whether a case progresses from mild to severe and countries with lower capacity health systems may thus, all else equal, experience a higher share of severe cases.

2) Adjusting the probability of dying given a severe case, $P_{Ias}(d|severe, c, inc_j)$, for health system capacity in a country in income group $inc_j$is the main contribution of Section 3. Health system capacity for a country in income group inc_j relative to France (and other high income countries) is proxied by the relative ability to treat childhood respiratory syncytial virus (RSV) ALRI. This is measured by the ratio of under-5 hospital infection fatality rates for RSV between income group inc_j and France, $\frac{hCFR(RSV<5|inc_j)}{hCFR(RSV<5|France)}$. To control for the different probabilities of reaching hospital in different income regions, we rescale this ratio by a factor equal to the ratio between childhood influenza IFRs in LMICs and France and the ratio of hospital IFRs in LMICs and France. The adjustment factor thus becomes:

$$adj_{inc_j} = \frac{hCFR(RSV < 5|inc_j)}{hCFR(RSV < 5|France)} * \frac{IFR(inf < 5|inc_j)}{IFR(inf < 5|France)} \Big/ \frac{hCFR(inf < 5|inc_j)}{hCFR(inf < 5|France)}\,.$$

We then calculate the (age and sex-dependent) odds of dying from a severe case of

COVID-19 in a country in income group j by multiplying the odds in France by the health system adjustment factor for income region j

$$\frac{P_{Ias}(d|severe, c, inc_j)}{1 - P_{Ias}(d|severe, c, inc_j)} = \frac{P_{Ias}(d|severe, c, France)}{1 - P_{Ias}(d|severe, c, France)} * adj_{inc_j} \quad \text{(A.3)}$$

and finally solve for

$$P_{Ias}\left(d\middle|severe, c, inc_j\right) = \frac{P_{Ias}(d|severe,c,France)*adj_{inc_j}}{1+P_{Ias}(d|severe,c,France)*\left(adj_{inc_j}-1\right)}. \quad \text{(A.4)}$$

Applying the adjustment to the odds of dying conditional on a severe case, rather than directly to the probability, ensures that the resulting probability is bounded between 0 and 1 and that the health system adjusted cIFR can never exceed the share of severe cases (conditional on age, sex and comorbidity).

Substituting $P_{Ias}(d|severe, c, inc_j)$ in equation (A.2), we obtain:

$$P_{Ias}\left(d\middle|\, c, inc_j\right) = \frac{P_{Ias}(d|severe,c,France)*adj_{inc_j}}{1+P_{Ias}(d|severe,c,France)*(adj_{inc_j}-1)} * P_{Ias}(severe|c). \quad \text{(A.5)}$$

Since we take $P_{Ias}(severe|c)$ to be a universal parameter, valid also for France, and assume mild cases to be self-limiting, $P_{Ias}(d|severe, c, France) * P_{Ias}(severe|c)$ equals the unadjusted cIFR we estimated in Section 2. (A.5) thus simplifies to:

$$P_{Ias}\left(d\middle|\, c, inc_j\right) = \frac{adj_{inc_j}}{1 + P_{Ias}(d|severe, c, France) * (adj_{inc_j} - 1)} * cIFR\,. \quad \text{(A.6)}$$

3) The final step calculates $P_{Ias}(d|severe, c, France)$, which from Bayes rule equals

$$P_{Ias}(d|severe, c, France) = \frac{P_{Ias}(c|d,severe,France)}{P_{Ias}(c|severe,France)} * P_{Ias}(d|severe, France)\,. \quad \text{(A.7)}$$

We retrieve the two factors in (A.7) in the following way:

3a) To calculate the second term on the right-hand side of (A.7), we assume that in high income countries such as France all severe cases obtain hospital care. $P_{Ias}(d|severe, France)$ can then be taken from [26] (p.37), which reports the probability of dying from COVID-19 in hospital in France.

3b) Before calculating the first term on the right-hand side of (A.7), note that in equation (1) in the main text, we do not condition on severity, and can recover the probability in the denominator of the Bayes' rule formula from the GBD data, for France or any other country. To our knowledge, data is not available from France to estimate the same probability conditional on severity. Instead, we calculate the ratio $\frac{P_{Ias}(c|d,severe,France)}{P_{Ias}(c|severe,France)}$ from two prior studies that report the presence of comorbidities among hospitalized patients in other HIC settings ([27] in NYC and [28] in Italy). The data in [27] covers 5700 patients hospitalized

for COVID-19 in a New York City hospital and reports at least one comorbidity for 94% of them. In addition, [28] analyses 411 patients with COVID-19 in an Italian hospital and reports comorobidity rates of 62% among hospitalized patients 84% among fatalities. To use this information, we assume that $P_{Ias}(c|severe)$ is independent of age and sex. That gives us a ratio of 1.05 for the prevalence of comorbidities among COVID-deaths to severe cases in New York City (combining the data in [7] and [27]) and a ratio of 1.35 for Italy.[1]

To calculate the health system adjusted cIFR, we set the ratio $\frac{P_{Ias}(c=1|d,severe,France)}{P_{Ias}(c=1|severe,France)} = \frac{99\%}{94\%} = 1.05.$ and, conversely, $\frac{P_{Ias}(c=0|d,severe,France)}{P_{Ias}(c=0|severe,France)} = \frac{1-99\%}{1-94\%} = 0.17.$ We test the sensitivity of the results to a higher ratio by also calculating the health system adjusted IFRs for $\frac{P_{Ias}(c=1|d,severe,France)}{P_{Ias}(c=1|severe,France)} = 1.35.$ and $\frac{P_{Ias}(c=0|d,severe,France)}{P_{Ias}(c=0|severe,France)} = 0.42,$ as in the Italian study. This reduces the IFR for LICs by 11% on average, by 9% for LMICs and by 7% for UMICs.[2]

[1] Although we cannot corroborate the assumption that the prevalence of comorbidities among severe COVID-19 cases is independent of age and sex, the numbers are close to the prevalence of comorbidities among COVID-19 deaths, for which we do have corroborating evidence for the independence assumption. In our view, this makes it reasonable to assume independence from age and sex also for the prevalence of comorbidities among severe COVID-19 cases.

[2] Detailed results available on request.

Figure A.1, Panel A shows model-based IFRs estimated by [26] for France and two separate estimates by [11] for Italy, the first using the adjusted Positive Test Rate (PTR) and the second calibrating them with the Diamond Princess IFRs. The IFR estimates in the two countries have very similar patterns, by age. We additionally report in Table A.1 the IFR by age and sex from [26], $P(d\,|I, age, sex)$, that we use as an input into our calculation.

Figure A.1, Panel B shows the percentage of the population with zero or any comorbidity by age and sex in France and Italy, and the ratio between the female and male population. The distributions are virtually identical. This gives us confidence in combining data from both countries for the estimation.

Figure A.1, Panel C shows the share of people who died with COVID-19 and any comorbidity in Italy (black line) and by sex and age group in New York City (bars and diamonds). The data is taken from Istituto Superiore della Sanità for Italy and New York City daily updates [7] on 16th May. Data for fatalities younger than 17 in NYC have been dropped in the age analysis as only nine deaths have been reported. The figure illustrates the very high presence of comorbidities among the COVID-19 fatalities for both sexes and in all age groups and the absence of a clear pattern by either of these variables.

Figure A.2 displays crude IFRs (in the first map) and IFRs adjusting for health-system capacity by country income group (in the second map) for all the countries in the GBD data. Darker color indicates higher values, the scale is common in both maps, such that it is possible to compare the crude and the health system adjusted IFR both within and across countries.

[FIGURE A.1 HERE]

Table A.1: IFRs used in our estimation

	IFR (%)	
Age	Female	Male
0-19	0.001	0.001
20-29	0.005	0.008
30-39	0.02	0.03
40-49	0.04	0.07
50-59	0.2	0.3
60-69	0.6	1.2
70-79	1.6	3.3
80+	5.9	17.1

Notes: Source [5]

[FIGURE A.2 HERE]
