# Supplementary figures and images for: Predicted COVID-19 fatality rates based on age, sex, comorbidities and health system capacity
Source: BMJ Glob Health. 2020 Sep 9;5(9):e003094. doi: 10.1136/bmjgh-2020-003094 (PMC7482102; doi:10.1136/bmjgh-2020-003094)

A: IFRs by country

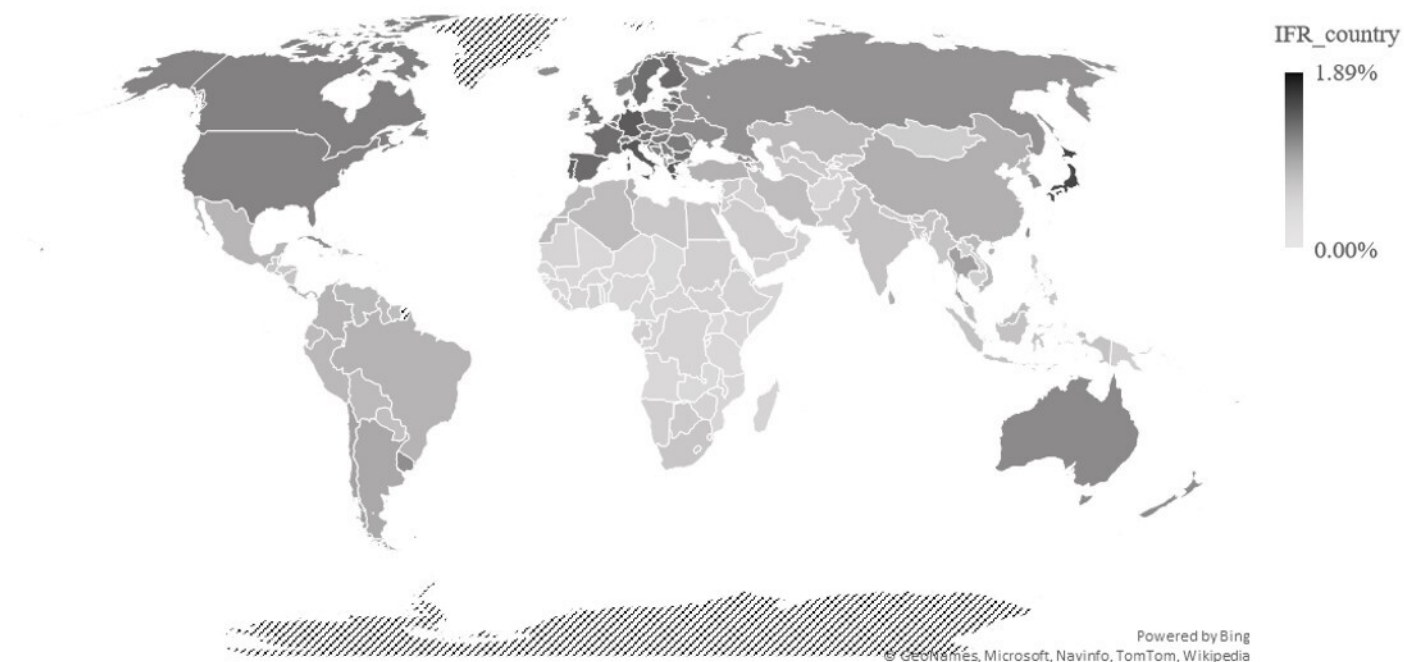

B: IFRs adjusted by health system capacity by country

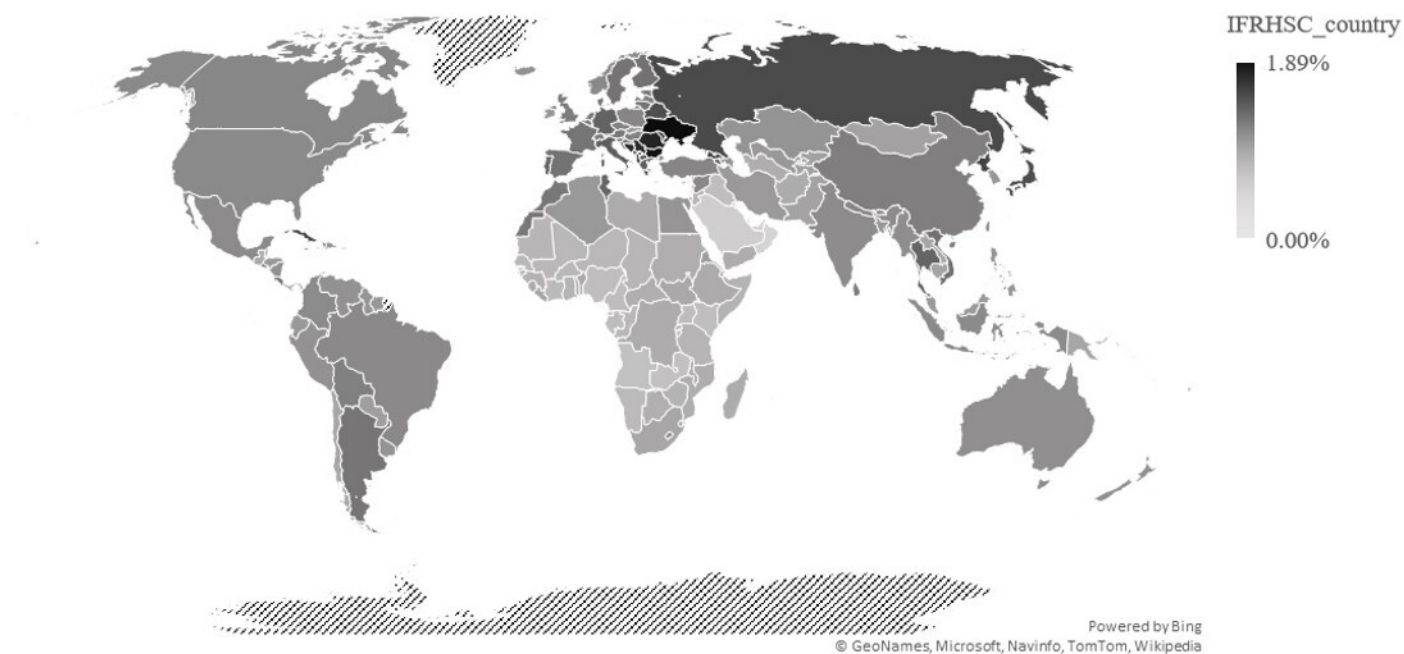

Supplement: Supplementary data [file bmjgh-2020-003094supp003.pdf]
